# Neuroprotective effect of dexmedetomidine on autophagy in mice administered intracerebroventricular injections of Aβ_25–35_

**DOI:** 10.3389/fphar.2023.1184776

**Published:** 2023-08-17

**Authors:** Youn Young Lee, Jong In Han, Kyung Eun Lee, Sooyoung Cho, Eun Cheng Suh

**Affiliations:** ^1^ Department of Anesthesiology and Pain Medicine, Ewha Womans University Seoul Hospital, Seoul, Republic of Korea; ^2^ Department of Anesthesiology and Pain Medicine, College of Medicine, Ewha Womans University, Seoul, Republic of Korea; ^3^ Department of Anesthesiology and Pain Medicine, Ewha Womans University Mokdong Hospital, Seoul, Republic of Korea; ^4^ Department of Pharmacology, College of Medicine, Ewha Womans University, Seoul, Republic of Korea

**Keywords:** dexmedetomidine, autophagy, Alzheimer’s disease, amyloid β-protein, behavioral test

## Abstract

Alzheimer’s disease (AD), one of the most prevalent neurodegenerative diseases is associated with pathological autophagy-lysosomal pathway dysfunction. Dexmedetomidine (Dex) has been suggested as an adjuvant to general anesthesia with advantages in reducing the incidence of postoperative cognitive dysfunction in Dex-treated patients with AD and older individuals. Several studies reported that Dex improved memory; however, evidence on the effects of Dex on neuronal autophagy dysfunction in the AD model is lacking. We hypothesized that Dex administration would have neuroprotective effects by improving pathological autophagy dysfunction in mice that received an intracerebroventricular (i.c.v.) injection of amyloid β-protein fragment 25–35 (Aβ_25–35_) and in an autophagy-deficient cellular model. In the Y-maze test, Dex reversed the decreased activity of Aβ_25–35_ mice. Additionally, it restored the levels of two memory-related proteins, phosphorylated Ca^2+^/calmodulin-dependent protein kinase II (p-CaMKII) and postsynaptic density-95 (PSD-95) in Aβ_25–35_ mice and organotypic hippocampal slice culture (OHSC) with Aβ_25–35_. Dex administration also resulted in decreased expression of the autophagy-related microtubule-associated proteins light chain 3-II (LC3-II), p62, lysosome-associated membrane protein2 (LAMP2), and cathepsin D in Aβ_25–35_ mice and OHSC with Aβ_25–35_. Increased numbers of co-localized puncta of LC3-LAMP2 or LC3-cathepsin D, along with dissociated LC3-p62 immunoreactivity following Dex treatment, were observed. These findings were consistent with the results of western blots and the transformation of double-membrane autophagosomes into single-membraned autolysosomes in ultrastructures. It was evident that Dex treatment alleviated impaired autolysosome formation in Aβ mice. Our study demonstrated the improvement of memory impairment caused by Dex and its neuroprotective mechanism by investigating the role of the autophagy-lysosomal pathway in a murine Aβ_25–35_ model. These findings suggest that Dex could be used as a potential neuroprotective adjuvant in general anesthesia to prevent cognitive decline.

## 1 Introduction

Alzheimer’s disease (AD) is one of the most prevalent neurodegenerative diseases associated with the progressive loss of synapses in the cerebral cortex and hippocampus, resulting in cognitive decline (DeKosky and Scheff, 1990). The pathological hallmarks of AD include extracellular accumulation of amyloid-beta (Aβ) protein and abnormally processed intraneuronal tau protein, also known as neurofibrillary tangles (NFTs) (Armstrong, 2009). Aβ is a major neurotoxic product of amyloid precursor protein (APP), which deposits into extracellular plaques in AD (Boland et al., 2018). Altered APP processing leads to Aβ accumulation, which could eventually cause AD (Nixon and Yang, 2011).

Autophagy is a principal mechanism for the degradation of abnormal and aggregated proteins, particularly under stress or injury conditions (Levine and Kroemer, 2008; Mizushima et al., 2008; Rubinsztein et al., 2015). It consists of sequential steps that degrade cytoplasmic substrates via the formation of autophagosomes and fusion with the lysosome (Mizushima et al., 2010). During autophagosome formation, a microtubule-associated protein called light chain 3 (LC3) and its lipidated form LC3-II lead to an elongating phagophore, which engulfs cytoplasm to form an autophagosome (Mizushima et al., 2010; Rubinsztein et al., 2015). P62, also called sequestosome 1 (SQSTM1), binds directly to LC3 (Jiang and Mizushima, 2015) and plays a role in the fusion of lysosomes with autophagosomes to form autolysosomes by establishing a bridge between LC3-II and ubiquitinated cargo (Pankiv et al., 2007).

Lysosome-associated membrane protein 2 (LAMP2), a heavily glycosylated type-1 lysosomal protein, is involved in the fusion of lysosomes with autophagosomes (González-Polo et al., 2005). Its deficiency leads to a defective fusion between the lysosome and autophagosome (González-Polo et al., 2005; Hubert et al., 2016). Subsequently, autophagosome cargo proteins are degraded by the lysosomal protease cathepsin D (Kaminskyy and Zhivotovsky, 2012). This process, termed “autophagic flux,” is the dynamic process of autophagosome synthesis, delivery of substrates to the lysosome, and degradation of autophagic substrates inside the lysosome (Mizushima et al., 2010). Accumulated autophagosomes either indicate increased autophagic induction or blocked maturation of autophagosomes in the lysosomal pathway (González-Polo et al., 2005; Mizushima et al., 2010). AD is a disease condition attributed to the progressive dysfunction of macroautophagy-mediated protein turnover in dystrophic neurites (Yu et al., 2005) that leads to impeded lysosomal degradation (Nixon and Yang, 2011).

Dexmedetomidine (Dex) is a highly selective α₂ adrenergic receptor agonist that is clinically used to induce perioperative analgesia with opioid-sparing effects or for sedation in patients in intensive care units (Keating, 2015). Unlike benzodiazepine, propofol, and opioids, Dex acts on sleep pathways at the locus coeruleus, independent of N-methyl-D-aspartate (NMDA) or gamma-aminobutyric acid A (GABA_A_) receptors (Huupponen et al., 2008), and has substantial benefits on respiratory and hemodynamic stability (Ebert et al., 2000). As the elderly population grows, so does the number of elderly people with or without degenerative brain disease who required anesthesia. Dex has several advantages, including being used as an adjuvant to general anesthesia to reduce the incidence of postoperative cognitive dysfunction and postoperative delirium (Zhou et al., 2016; Xin et al., 2021).

Previous studies have demonstrated the neuro- (Luo et al., 2017; Zhang et al., 2021) or organo- (Zhao et al., 2020; Zhu et al., 2020; Kho et al., 2022) protective mechanism of Dex by regulating autophagic mechanisms; however, its protective mechanism is controversial. Some studies showed that Dex upregulated the autophagy pathway in the neuroinflammatory microglial model (Zhang et al., 2021), while others demonstrated that Dex protected the brain from ischemia-perfusion injury via autophagy inhibition (Luo et al., 2017). Researchers focused on the underlying mechanism of the neuroprotective effects of Dex using different models because its impact on memory improvement showed consistent results. However, there is a lack of evidence regarding the neurocognitive effects of Dex in the elderly using the AD model. Previously, Sun et al. (2020) suggested that Dex protects hippocampal neuron apoptosis in AD mice; however, the neuroprotective mechanism of Dex in AD is still poorly understood. To the best of our knowledge, a few studies showed the influence of Dex on memory impairment in the AD model via an autophagy-lysosomal pathway.

As a result, we hypothesized that Dex might have neuroprotective effects on AD via the improvement of autophagic dysfunction. To identify the neuroprotective mechanism of Dex, we established murine Aβ_25–35_ models both *in vivo* and in organotypic hippocampal slice culture (OHSC), conducted behavioral tests, and analyzed memory- and autophagy-related proteins in the AD hippocampus.

## 2 Materials and methods

### 2.1 Experimental animals and intracerebroventricular (i.c.v.) injection of Aβ_25–35_

A total of 137 C57BL/6N mice (weight, 25 ± 3 g; age, 8–10 weeks) were purchased from DBL Co., Ltd. (Umsung-gun, Korea). Animals were kept in plastic cages and given *ad libitum* access to water and food under a 12 h light-dark cycle (lights on from 07:00 to 19:00). The cages were individually ventilated and housed in a pathogen-free facility.

For i.c.v. injection, mice were anesthetized using isoflurane (1.6%–2%) (Hana Pharm Co., Seoul, Korea) and placed in a stereotaxic apparatus. A Hamilton syringe attached to a Nanomite injector syringe pump (Harvard Apparatus, Holliston, MA, United States) was used for i.c.v. injection of Aβ_25–35_ (10 nM/3 μl per mouse) or saline (3 μl) at a rate of 1 μl/min, using the following coordinates: anteroposterior, −0.3 mm from the bregma; mediolateral, −1.0 mm from the midline; and depth, 2.5 mm from the skull (Yin et al., 2015; Kim et al., 2016). The needle of the microsyringe was kept in the hole for 3 min to ensure the proper injection of Aβ_25–35_ or saline. The mice that underwent surgery were kept in the rodent intensive care unit until they were awoken. This animal study was approved by the Institutional Animal Care and Use Committee (IACUC) of Ewha Womans University (Approval No. EWHA MEDIACUC 21-010-t). All animal procedures were performed in strict accordance with the Guide for the Care and Use of Laboratory Animals (National Institutes of Health, United States).

### 2.2 Dosage regimen and experimental group

After 2 weeks of adaptation from i.c.v. injection, we conducted a preliminary investigation to determine the Dex (Sigma-Aldrich, Darmstadt, Germany) dosage regimen. According to the references (Liu J. R. et al., 2016; Pan et al., 2016), Dex was still safe or neuroprotective at the dose of 320 μg/kg in mice, and 125 μg/kg in rat. So, we tested different intraperitoneal (IP) Dex doses ranging from 20 μg/kg to 240 μg/kg to determine clinically relevant sedative and hypnotic doses by measuring the loss of righting reflex response (LORR). LORR was used to confirm the response after placing the mice in the supine position. The LORR grading scale ranged from 0 to 4: 0 = no response, 1 = delayed attempt to right itself but failing, 2 = delayed, uncoordinated return to the upright position, 3 = sedated but righting themselves in a coordinated fashion, or 4 = awake, not remaining supine (Lee et al., 2021). The dosage below 40 μg/kg of Dex did not produce sedation or hypnosis. 40 μg/kg of Dex showed sedation (LORR score grade of 2 or 3) for 30 min, and 80 μg/kg of Dex showed hypnosis (LORR score grade of 0 or 1) for 30 min and full recovery.

**FIGURE 1:**
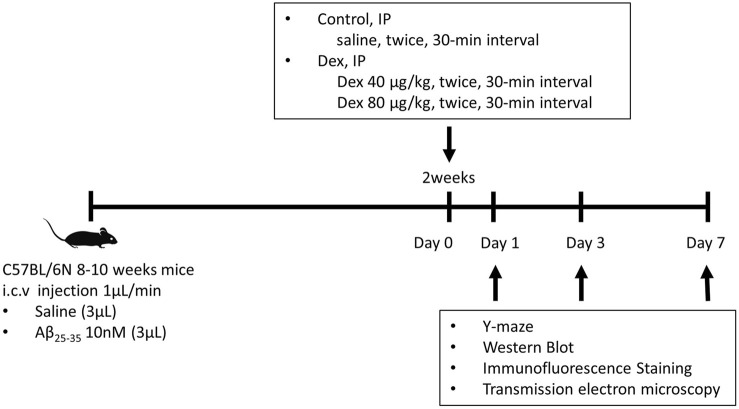
The protocol of Dex treatment in Aβ_25–35_ mice. A total of 137 C57BL/6N mice were injected with saline or Aβ_25–35_ intracerebroventricularly. Two weeks after the surgery, these mice were injected with Dex (40 μg/kg or 80 μg/kg) or saline intraperitoneally twice at 30 min intervals. The Y-maze behavior test was conducted on days 1, 3, and 7 following IP injection. Following the behavioral test, the brain was removed and divided into the left hemisphere and right hippocampus for Western blotting and immunofluorescence staining, respectively. i.c.v., intracerebroventricular; Aβ_25–35_, amyloid β-protein fragment 25–35; Dex, dexmedetomidine; IP, intraperitoneal.

The anesthetic dosage regimen was finally decided on as follows: IP injection of 40 μg/kg Dex was considered a sedative dose, whereas 80 μg/kg was considered a hypnotic dose. After 30 min, the mice were reinjected with the same amount of saline or Dex to prolong the sedation or hypnosis for an hour (Figure 1).

The mice were allocated to six groups according to the type of drug: control (saline i.c.v. + saline IP, *n =* 23); D40 (saline i.c.v. + Dex 40 μg/kg, *n =* 21); D80 (saline i.c.v. + Dex 80 μg/kg IP, *n =* 24); Aβ (Aβ_25–35_ i.c.v. + saline IP, *n =* 25); Aβ/D40 (Aβ_25–35_ i.c.v. + Dex 40 μg/kg IP, *n =* 25); and Aβ/D80 groups (Aβ_25–35_ i.c.v. + Dex 80 μg/kg IP, *n =* 21).

### 2.3 Y-maze test

To investigate the effects of short-term memory, relevant to postoperative cognitive impairment, a Y-maze test was conducted. For testing spontaneous alteration of behavior in the Y-maze test, an index of spatial working memory (Kraeuter et al., 2019), a total of 139 mice were examined on days 1, 3, and 7 following Dex injection (*n* = 43, 42, and 54, respectively). Prior to starting the behavior test, a testing mouse was adapted in the testing room for 30 min. Each mouse was tested at the same time for 10 min, starting at the center of the maze equipment. The testing maze consists of three black arms (30 × 6 × 15 cm) with 120° angles designated A, B, and C. We measured the number of consecutive arm entries and counted spontaneous alternations when the mice explored previously unvisited arms. For example, AAB or BCB were not counted; only three different arm entries, such as ABC or CAB, were counted as alternations. Spontaneous alternation behavior was calculated as the percentage of alternations versus total alternation opportunities (total arm entries minus two) (Arendash et al., 2001).

### 2.4 Protein extraction and western blot analyses

Following behavioral tests, the left hippocampus was dissected immediately and lysed in ice-cold lysis buffer (NP40 cell lysis buffer, ThermoFisher, Carlsbad, CA, United States) with a protease inhibitor (Roche Diagnostics GmbH, Penzberg, Germany). Protein concentrations were determined using the Bradford assay. Equal amounts of protein samples were loaded and transferred onto blotting membranes (0.2 μm nitrocellulose membrane; Bio-Rad Laboratories, Inc., CA, United States). After being blocked in Tris-buffered saline and Tween containing 10% skim milk for 1 h, the membranes were incubated with primary antibodies (Supplementary Table S1) overnight at 4°C, followed by incubation with secondary antibodies for 2 h (Supplementary Table S2). Immunoreactive bands were visualized with a luminescent image analyzer (ImageQuant LAS 4000 mini, GE Healthcare, Japan) using a chemiluminescence reagent (Amersham ECL Prime, GE Healthcare, Buckinghamshire, United Kingdom). Densitometric analysis of the bands was performed using the ImageJ software v. 1.8.0 program from the National Institutes of Health (NIH). The cultured slices in OHSC were also processed in the same way as described above.

The total number of mice in the Western blot was 135 of p-tau, 137 of PSD-95, 121 of p-CaMKII, 113 of LC3-II, 137 of p62, 137 of LAMP2, and 119 of cathepsin D. An average of 6–8 mice was used for each group on day 1, 3, and 7, respectively. The number of Western blot in OHSCs was conducted 5 to 9 times per group.

### 2.5 Immunofluorescence staining

Following the behavior test, the right hemisphere was fixed for 48 h in 0.1 M phosphate-buffered (pH 7.4) 4% paraformaldehyde (Merck; Darmstadt, Germany) and stored at 4°C in a 30% sucrose solution. A cryocut microtome (Leica CM 1850; Wetzlar, Germany) was used to make 30 μm cryosections of brain tissue. Cryosections were incubated for 1 h in a blocking solution containing 0.2% Triton X-100% and 10% bovine serum albumin in phosphate-buffered saline (PBS). The section was incubated with primary antibodies overnight, followed by incubation with secondary antibodies for 2 h (Supplementary Tables S1, S2). Images were obtained using a confocal microscope (ZEISS LSM 800, Zeiss; Jena, Germany). For each treatment condition, the numbers of immunofluorescence staining dots were counted using confocal microscopic images at a magnification of ×800 (80 μm^2^). The ImageJ software was used to determine the number of immunopositive dots.

### 2.6 Ultrastructural analysis with transmission electron microscopy (TEM)

The mouse brain was coronal sectioned (between −1.3 and −2.5 mm from bregma), and the CA3 area of the hippocampus was cut into small pieces (1 × 1 × 1 m^3^) and fixed with 2.5% glutaraldehyde in 0.1 M phosphate buffer (pH 7.4), postfixed in 1% osmium tetroxide (Ted Pella, Inc.; Redding, CA) for 1 h, gradually dehydrated in ethanol, and then embedded with Epon 812 resin (Electron Microscopy Sciences; Hartfield, PA). Semi-thin sections (100 nm) were cut from the tissue blocks and stained with 0.5% toluidine blue. Ultrathin sections were stained with 0.25% lead citrate and 2% uranyl acetate and were then observed with an electron microscope (Hitachi H7650, Hitachi; Tokyo, Japan). All of the images were captured on a Morada camera (Soft Imaging System, Olympus Soft Imaging Solutions GmbH; Münster, Germany) using charge-coupled device camera system software with an analysis program.

### 2.7 Organotypic hippocampal slice culture (OHSC) and drug treatments

The brains of the 8-day-old Sprague–Dawley rats were immersed in an ice-cold dissecting medium. Slices of hippocampi (400 μm) were made with a McIlwain tissue chopper (Mickle Laboratory Engineering; Surrey, United Kingdom). The transverse slices were placed on semi-porous Millicell membrane inserts (0.4 μm, Millicell-CM, Millipore; Bedford, MA, United States) and transferred to a 37°C, 5% CO_2_ incubator in 6-well culture plates (Falcon, Becton Dickinson; Franklin Lakes, NJ, United States) containing neurobasal culture medium (Gibco BRL; Grand Island, NY, United States). The medium was changed twice weekly for 3 weeks until the experiments commenced (Suh et al., 2008).

Aβ_25–35_ (Sigma-Aldrich; St. Louis, MO, United States) was prepared as a 1 mM stock solution in sterile deionized water and then incubated at 37°C for 24 h to obtain the aggregated form. The hippocampal slices were grown in a medium supplemented with 2.5 μM Aβ_25–35_ for 3 days (Cho et al., 2018). Dex was added to Aβ_25–35_ in a culture medium at final concentrations of 1 or 2.5 μM, according to the propidium iodide uptake experiments. To evaluate the effect of Dex on the autophagy flux, bafilomycin A1 (BafA1, 10 nM) was added to the culture medium with or without Dex. BafA1 is a reversible, potent and specific inhibitor of the vacuolar type H (^+^)-ATPase (V-ATPase) in cells, and blocks the fusion of autophagosomes with lysosomes. (Klionsky et al., 2021).

### 2.8 Propidium iodide (PI) uptake in OHSCs

The extent of neuronal cell death was assessed using the fluorescent exclusion dye, propidium iodide (PI) (Suh et al., 2008; Cho et al., 2018). PI was added (2 μg/ml) to the culture medium and incubated in the dark for 1 h at 37°C. Following the washing of PBS, images were obtained using an inverted fluorescence microscope (Zeiss Axiovert 200, Zeiss, Göttingen, Germany). The image intensity was analyzed using the Axio Vision v. 4.7.1.0 program (Imaging Solutions GmbH, Munich, Germany).

### 2.9 Statistical analysis

The results are presented as means ± standard error of mean (SEM). The data were analyzed using one-way analysis of variance (ANOVA) using StatView v.5 software (SAS Institute, Inc., Cary, NC, United States). Fisher’s post-hoc test was used to determine means that were significantly different from the control mean. Statistical significance was set at *p* < 0.05.

## 3 Results

### 3.1 Dex decreased p-tau in Aβ_25–35_ mice

**FIGURE 2:**
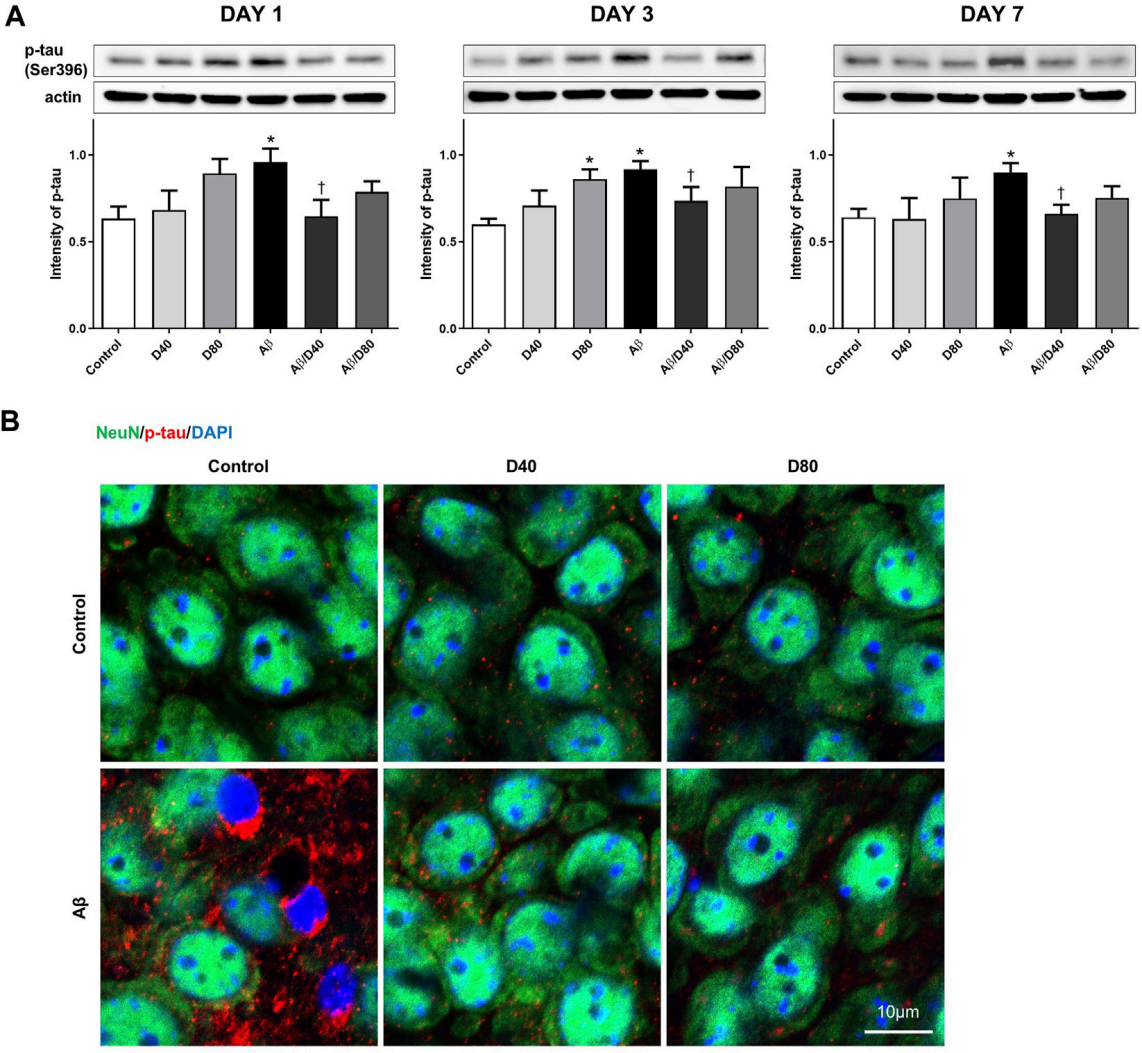
Western blot analyses and immunofluorescence staining of p-tau following treatment with Dex or saline intraperitoneally **(A)** A significant increase in the intensity of p-tau was observed in the Aβ group compared to that in the control on days 1, 3, and 7. The intensity of p-tau was reversed in the Aβ/D40 group compared to that in the Aβ group on days 1, 3, and 7. Data are presented as the mean ± standard error of mean. *p < 0.05, compared to the control; ^†^ *p* < 0.05, compared to the Aβ group. **(B)** Confocal microscopic images of p-tau at the pyramidal cell layer of CA3 in the hippocampus following treatment with intraperitoneal Dex or saline on day 7. While increased p-tau in chromatin condensation was observed in the Aβ group, a significant decrease in p-tau was observed in the Aβ/D40 and Aβ/D80 groups. Scale bar: 10 μm.

To establish the Aβ model, we confirmed p-tau intensity in Aβ mice using Western blotting and immunofluorescence staining. A significant increase in the intensity of p-tau was observed in the Aβ group compared to that in the control group on days 1, 3, and 7 (Figure 2A). Increased p-tau levels in the Aβ group from the immunofluorescence image on day 7 (Figure 2B) confirmed the Aβ modeling is proper. The intensity of p-tau was significantly reduced in mice from the Aβ/D40 group compared to those from the Aβ group on days 1, 3, and 7. The Aβ/D80 group showed decreasing tendency in p-tau level which was not significant. Mice treated with only D40 and D80 showed no statistical difference compared to the control, except for a significant increase in p-tau level in the D80 group on day 3 (Figure 2A). Confocal microscopic images revealed decreased p-tau levels following Dex administration (40 μg/kg, 80 μg/kg) on days 1, 3 (data not shown), and 7 (Figure 2B), indicating that Dex reduced p-tau production.

### 3.2 Dex reversed the result of the Y-maze test in Aβ_25–35_ mice

**FIGURE 3:**
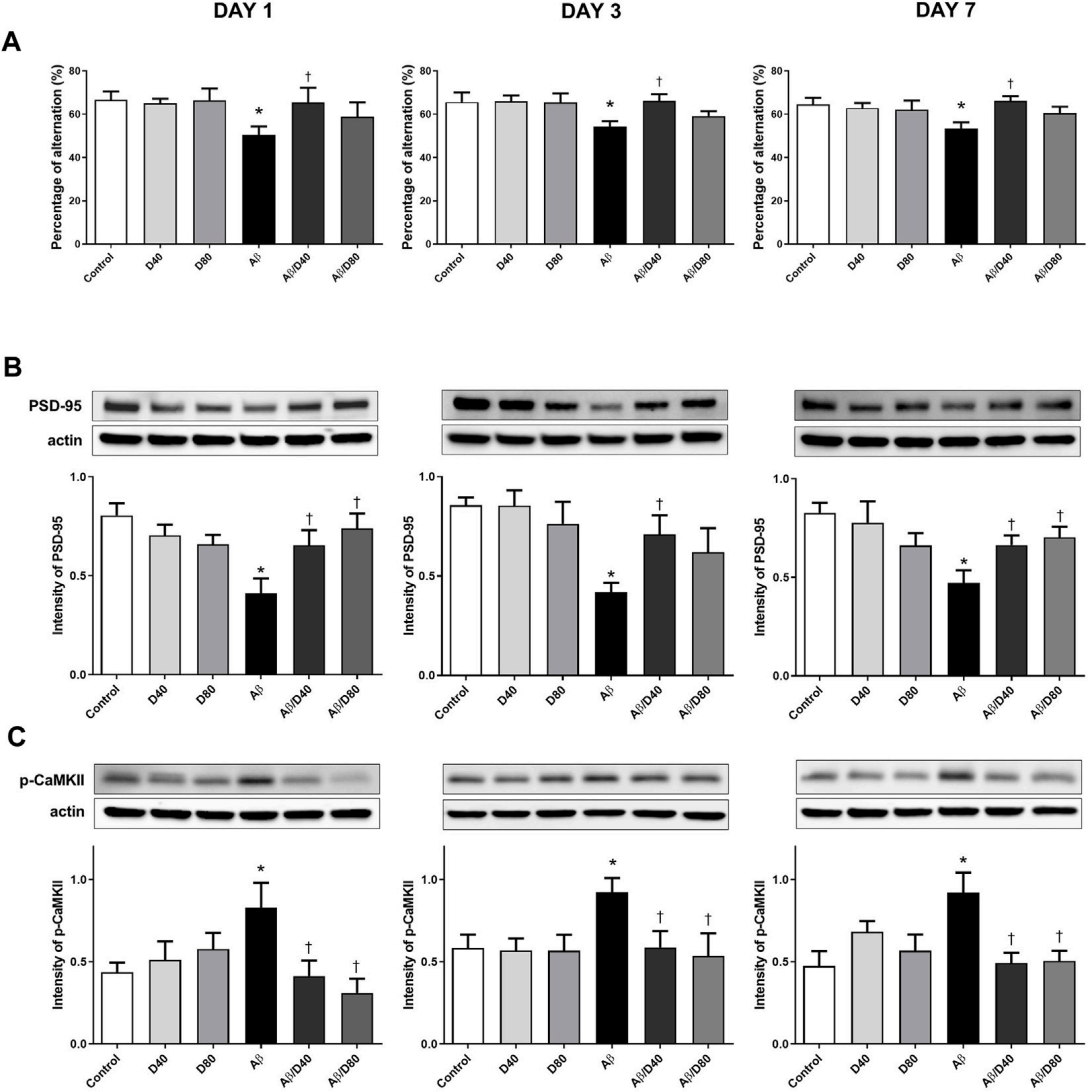
The result of Y-maze tests and Western blot analyses of PSD-95 and p-CaMKII on days 1, 3, and 7 after treatment with Dex or saline intraperitoneally **(A)** A significant decrease in the percentage of alternation was observed in the Aβ group compared to that in the control, and a significant recovery of the behavior was observed in the Aβ/D40 group on days 1, 3, and 7. There was no significant difference in the results among the saline ic.v. groups (control, D40, and D80). **(B)** On days 1, 3, and 7, the intensity of PSD-95 levels decreased significantly in the Aβ group compared to those in the control. The reversed intensity of PSD-95 was observed in the Aβ/D40 group on days 1, 3, and 7. **(C)** The intensity of p-CaMKII levels increased substantially in the Aβ group compared to those in the control on days 1, 3, and 7. A significant decrease in the intensity of p-CaMKII was observed in the Aβ/D40, and Aβ/D80 groups compared to that in the Aβ group on days 1, 3, and 7. Data are presented as the mean ± SEM. *p < 0.05, compared to the control, ^†^ *p* < 0.05, compared to the Aβ group.

The behavior analysis of spatial learning memory was performed using the Y-maze test to evaluate whether Dex improved memory impairment in Aβ mice. In the Aβ group, there were significant decreases in the spontaneous behavior activity compared to the control on days 1, 3, and 7, and in Aβ/D40 group, the decreases were recovered significantly almost to the levels of the control at all time points (days 1, 3, and 7). (Figure 3A). The recovered behavior activity in the Aβ/D80 group compared to the Aβ group on days 1, 3, and 7, showed some tendencies with no significant difference. There were no differences in behavioral performance between the Dex-only treated groups (D40 and D80) and the control group.

### 3.3 Dex-attenuated dysregulation of PSD-95 and p-CaMKII is associated with reversed memory function in Aβ_25–35_ mice

The levels of PSD-95 and p-CaMKII were analyzed to investigate the effect of Dex on memory-related proteins in Aβ mice (Figures 3B, C). The PSD-95 levels in the Aβ group decreased significantly compared to the control on days 1, 3, and 7 (Figure 3B). Dex significantly reversed PSD-95 levels in Aβ/D40 mice compared to the Aβ group on days 1, 3, and 7 and in Aβ/D80 mice on days 1 and 7, indicating that administering Dex restored Aβ-induced memory deficit. The decreasing tendency of PSD-95 levels was demonstrated in the D80 group compared to the control, but there were no significances.

The level of p-CaMKII increased in the Aβ group compared to the control on days 1, 3, and 7 (Figure 3C). Aβ/D40 and Aβ/D80 mice showed a significant decrease in the intensity of p-CaMKII on days 1, 3, and 7. Restoring p-CaMKII to the control level after administering Dex in Aβ/D40 and Aβ/D80 groups showed recovered memory impairment in the Aβ group. The Dex-only treated groups (D40 and D80) did not differ substantially from the control. Overall, the results of the behavior test and memory-related proteins suggested that Dex mitigated memory dysfunction induced by Aβ_25–35_ toxicity.

### 3.4 Dex attenuated the impaired autophagic flux in Aβ_25–35_ mice

To evaluate the effect of Dex on the Aβ_25–35_-induced impaired autophagic pathway, we analyzed changes in autophagy-related proteins: LC3-II, p62, LAMP2, and cathepsin D.

**FIGURE 4:**
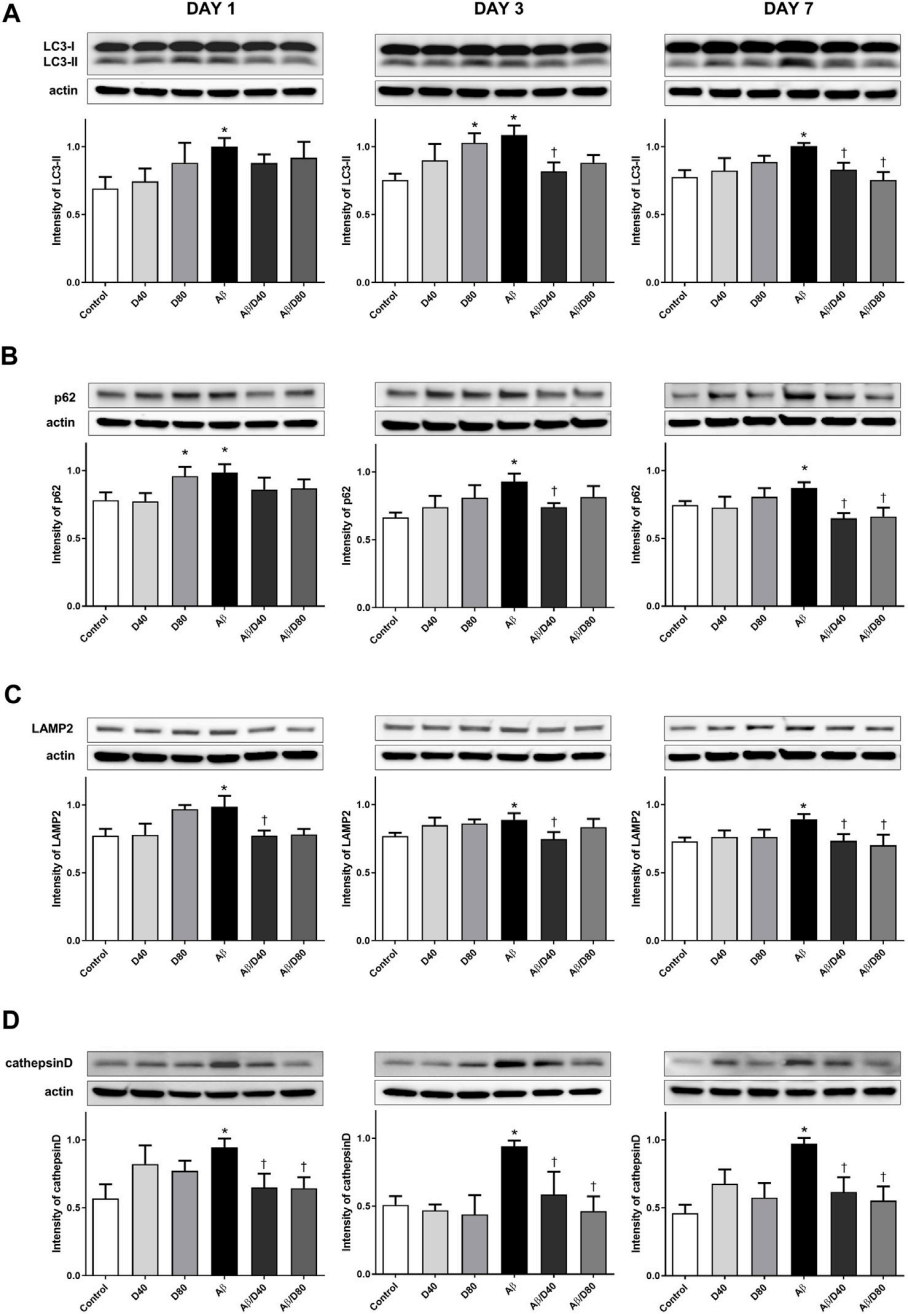
Western blot analyses of LC3-II, p62, LAMP2, and cathepsin D following treatment with intraperitoneal Dex or saline **(A)** The intensity of LC3-II levels increased significantly in the Aβ group compared to that in the control on days 1, 3, and 7. After Dex treatment, the expression levels of LC3-II were significantly reduced in the Aβ/D40 group on days 3 and 7, and in the Aβ/D80 group on day 7. **(B)** A significant increase in the intensity of p62 was observed in the Aβ group compared to that in the control on days 1, 3, and 7. The intensity of p62 levels decreased significantly in the Aβ/D40 group on days 3 and 7, and in the Aβ/D80 group on day 7 **(C)** A significant increase in the intensity of LAMP2 was observed in the Aβ group compared to that in the control on days 1, 3, and 7. The intensity of LAMP2 levels reversed significantly in the Aβ/D40 on days 1, 3, and 7. **(D)** Cathepsin D levels increased considerably in the Aβ group compared to that in the control on days 1, 3, and 7. The intensities of cathepsin D were significantly reduced in the Aβ/D40 and Aβ/D80 groups on days 1, 3, and 7. Data are presented as the mean ± SEM. *p < 0.05, compared to the control, ^†^ *p* < 0.05, compared to the Aβ group.

There were significant increases in the levels of LC3-II in the Aβ group compared to the control on days 1, 3, and 7 (Figure 4A). Increases in the LC3-II levels indicated the increased formation of the autophagosome, possibly under strong autophagy induction or impaired autophagic clearance conditions (Nixon and Yang, 2011). In AD, strongly induced autophagosome formation could exacerbate already made Aβ and toxic metabolites because autophagosome clearance may be impaired (Boland et al., 2008). After Dex treatment, the expression levels of LC3-II were significantly reduced in the Aβ/D40 group on days 3 and 7, and in the Aβ/D80 group on day 7 (Figure 4A). This result indicated that accumulated substrates were degraded by improving autophagosome clearance after Dex administration. The intensities of LC3-II in the D80 group on day 3 increased significantly compared to the control.

The levels of p62 in the Aβ group increased significantly on days 1, 3, and 7 (Figure 4B). Administrating Dex to Aβ mice significantly reversed the increased intensity of p62 in the Aβ/D40 group on days 3 and 7, and in the Aβ/D80 group on day 7. The intensity of p62 in the D80 group increased significantly on day 1 compared to the control.

**FIGURE 5:**
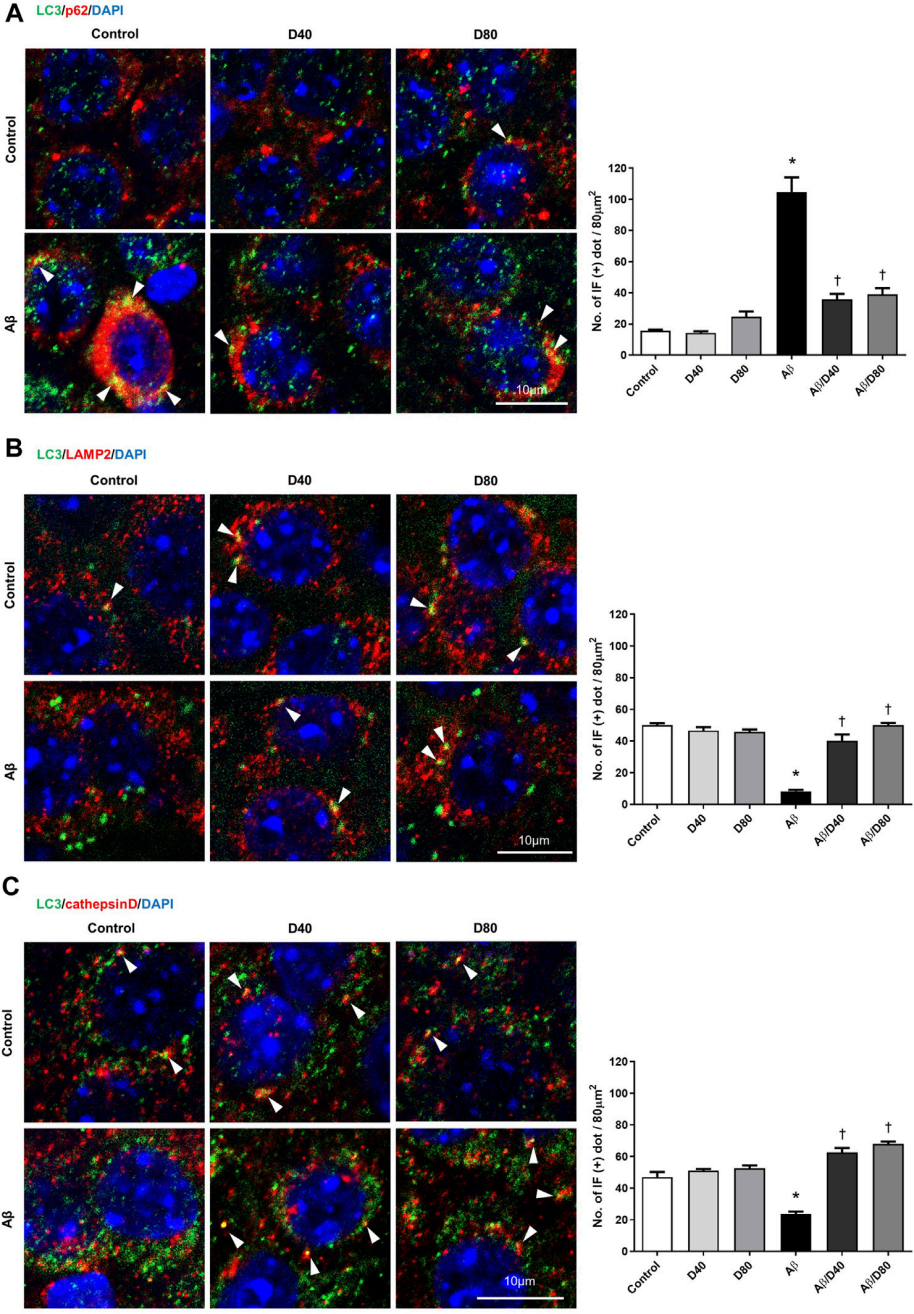
Confocal microscopic images of LC3, p62, LAMP2, and cathepsin D at the pyramidal cell layer of CA3 in the hippocampus following treatment with intraperitoneal Dex or saline on day 7 **(A)** LC3/p62/DAPI triple confocal microscopic images. Confocal images demonstrated that LC3/p62 co-localized puncta (yellow dots, white arrowhead). The number of LC3/p62 immunofluorescence dots per 80 μm^2^ was presented in the graph. There were significantly fewer puncta in the co-localized immunoreactivity of LC3 and p62 in the Aβ/D40 and Aβ/D80 groups than in the Aβ group. **(B)** LC3/LAMP2/DAPI triple confocal microscopic images. Confocal images revealed that LC3/LAMP2 co-localized puncta and that LC3 and LAMP2 puncta increased in the Dex treatment groups (white arrowhead). The number of LC3/LAMP2 immunofluorescence dots per 80 μm^2^ was presented in the graph. There were significantly more puncta in the co-localized immunoreactivity of LC3 and LAMP2 in the Aβ/D40 and Aβ/D80 groups compared to those in the Aβ group. **(C)** LC3/cathepsin D/DAPI triple confocal microscopic images. The co-localized immunoreactivity of LC3 and cathepsin D decreased in the Aβ group compared to that in the control and increased in the Aβ/D40 and Aβ/D80 groups compared to that in the Aβ group (white arrowhead). The number of LC3/cathepsin D immunofluorescence dots per 80 μm^2^ was presented in the graph. There were significantly more puncta in the co-localized immunoreactivity of LC3 and cathepsin D in the Aβ/D40 and Aβ/D80 groups compared to those in the Aβ group. Quantification of the number of immunofluorescent dots was performed using the ImageJ quantification tool. The significances indicate differences from control values (*) or Aβ_25–35_ treatment (^†^) (*p* < 0.05, one-way analysis of variance; ANOVA on ranks followed by Fisher’s PLSD method). Scale bar: 10 µm.

In immunofluorescence staining, co-localized puncta of LC3/P62 were significantly increased in the Aβ group (Figure 5A), indicating increased induction of autophagosome formation. Accumulated co-localized puncta in the Aβ group decreased significantly in the Aβ/D40 and Aβ/D80 groups on day 7, consistent with Western blot analysis (Figure 5A). This result suggested that Dex promoted autophagosomal degradation by the lysosome, indicating a progression of balance between autophagosome formation and clearance by the lysosome (Chu, 2006).

LAMP2 levels were significantly increased in the Aβ group compared to the control group, and significantly decreased in the Aβ/D40 group to the control level on days 1, 3, and 7, and in the Aβ/D80 group on day 7 (Figure 4C). There were no differences between the control group and Dex-only treated group (D40 or D80). In immunofluorescence staining, co-localized puncta of LC3/LAMP2 were significantly decreased in the Aβ group, and these were reversed after Dex treatment in the Aβ/D40 and Aβ/D80 groups (Figure 5B), indicating Dex improved fusion between autophagosomes and lysosomes to form autophagolysosomes.

Consistent with the result of LAMP2, increased levels of cathepsin D in the Aβ group significantly decreased in the Aβ/D40 and Aβ/D80 groups on days 1, 3, and 7 (Figure 4D). In immunofluorescence staining, co-localized punta of LC3/cathepsin D were significantly decreased in the Aβ group, and these were significantly reversed in the Aβ/D40 and Aβ/D80 groups (Figure 5C). Overall, these findings indicated that Dex promoted autophagosome formation, in addition to leading to autolysosomal degradation in the late stages, promoting the entire autophagy flux.

### 3.5 Dex improved impaired autophagic flux by transforming autophagosome to autolysosome

**FIGURE 6:**
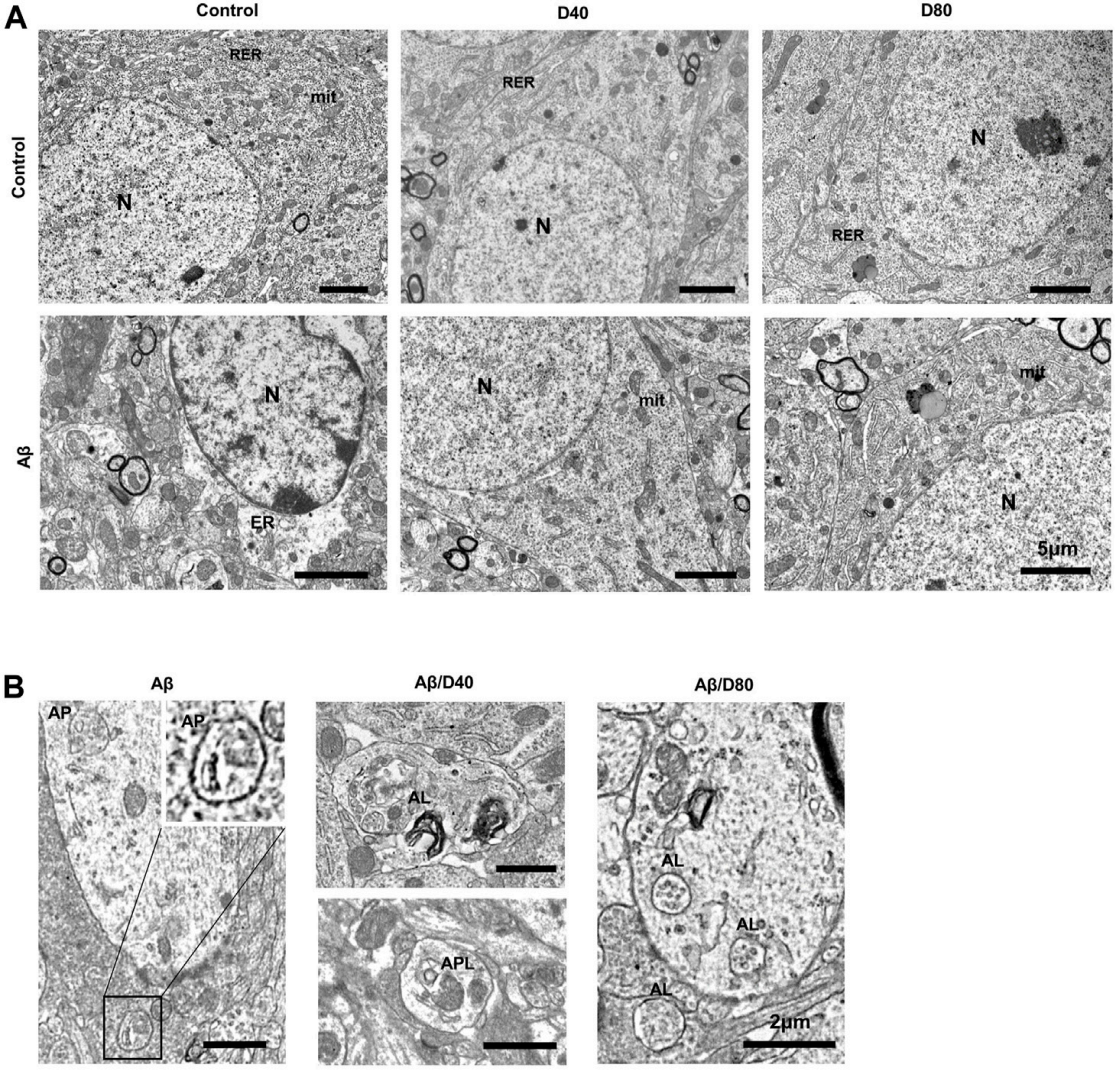
The ultrastructural images of pyramidal neurons in the CA3 region in the hippocampus of C57B/6N mice on day 7 following Dex treatment, after i.c.v. injection of Aβ_25-35_ or saline **(A)** The ultrastructure of neurons in control showed well-organized nucleus (N), mitochondria (mit), and endoplasmic rough reticulum (RER). The D40 or D80 group had no differences compared to the control. The Aβ group showed rarefaction of cytosol, dilatation of the endoplasmic reticulum (ER) and nuclear chromatin agglutinates. The Aβ/D40 and Aβ/D80 groups were morphologically intact, similar to the control. (scale bar = 5 μm) **(B)** Magnified images of the autophagic vacuoles (AVs) after i.c.v. injection of Aβ_25-35_. In the Aβ group, A0056s observed were mostly double-membraned AVs, morphologically similar to autophagosomes (AP). In the Aβ/D40 and Aβ/D80 groups, single-membraned AVs (autolysosome, AL) were mainly observed and some autophagolysome (APL), autophagosomes combined with lysosomes, were also noticed, suggesting improvement of autophgic flux by Dex treatment. (scale bar = 2 μm).

The ultrastructure of the pyramidal neurons showed a well-organized nucleus, mitochondria, and endoplasmic rough reticulum, indicating healthy neurons in all groups except the Aβ group. The Aβ group showed rarefaction of cytosol, dilatation of the endoplasmic reticulum, and nuclear chromatin agglutinates (Figure 6A). In the magnified images, double-membraned autophagosomes are mainly observed in the Aβ group, suggesting impaired autophagic flux by Aβ_25-35_. On the other hand, single-membraned autolysosomes are mainly observed in the Aβ/D40 or Aβ/D80 groups, suggesting improvement of autophagic flux by Dex treatment (Figure 6B).

### 3.6 Dex attenuated Aβ_25–35_-induced cell toxicity and impaired autophagic flux in OHSCs

**FIGURE 7:**
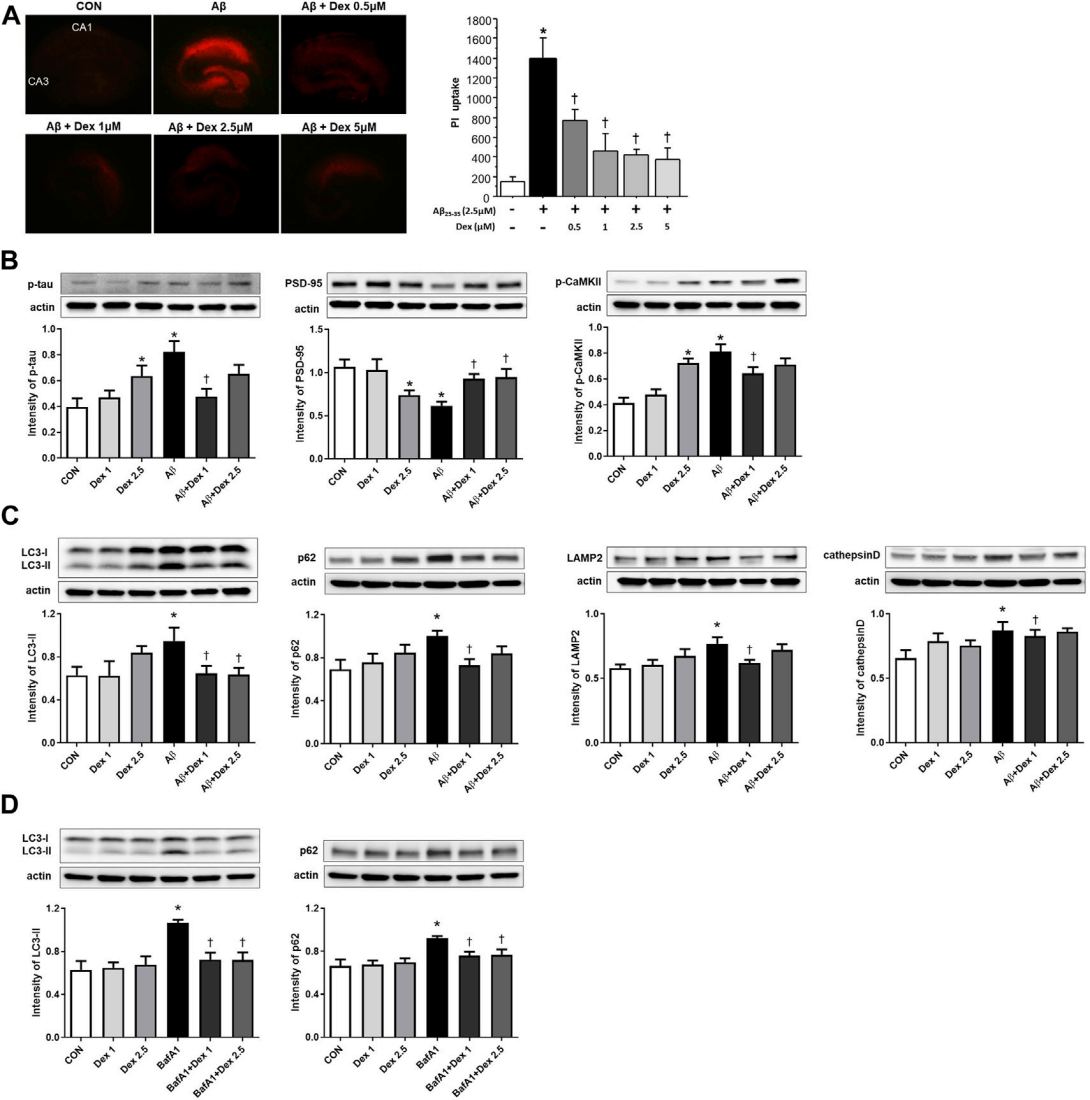
Detection of neuronal cell death using propidium iodide (PI) uptake and Western blot analysis in organotypic rat hippocampal slice cultures (OHSCs). **(A)** Representative images showing PI uptake in the control, only Aβ_25–35_ treatment, and co-treated with the four different concentrations (0.5, 1, 2.5, and 5 μM) of Dex groups. The PI uptake in the Aβ_25–35_-only-treated solution was significantly higher than in the control and decreased as the concentration of Dex in Aβ_25–35_ increased. **(B)** The intensities of p-tau (*n* = 53) and p-CaMKII (*n* = 37) significantly increased in OHSCs treated with Aβ_25–35_-only media and decreased significantly in the cultures treated with Dex (1 μM) and Aβ_25–35_. The intensities of PSD-95 (*n* = 54) showed a significantly decreased level in OHSCs treated with Aβ_25–35_ only media and an increased level in the cultures treated with Dex (1 and 2.5 μM) and Aβ_25–35_. **(C)** The intensities of LC3-II (*n* = 30), p62 (*n* = 42), LAMP2 (*n* = 36), and cathepsin D (*n* = 36) increased significantly in OHSCs treated with Aβ_25–35_ only compared to those in the control. The intensity of LC3-II, p62, LAMP2 and cathepsin D decreased significantly after administration of Dex (1 μM) to Aβ_25–35_. **(D)** Western blot analyses of LC3-II and p62 following treatment Dex and bafilomycin A1 (BafA1, 10 nM). The intensities of LC3-II (*n* = 36) and p62 (*n* = 36) increased significantly in OHSCs treated with BafA1 compared to control. The intensity of LC3-II and p62 decreased significantly to the control level after administration of Dex to BafA1. Data were presented as the mean ± standard error of mean. *p < 0.05 compared to the control, ^†^ *p* < 0.05 compared to the group treated with only Aβ_25–35_.

Cell viability after Dex treatment was observed to further validate the neuroprotective effect of Dex in OHSC. PI uptake was increased in OHSCs with Aβ_25–35_ compared to the control, which indicated an increase in neuronal cell death. Administration of Dex at four different concentrations (0.5, 1, 2.5, and 5 μM) to Aβ_25–35_-treated OHSCs resulted in significantly decreased uptake of PI in the pyramidal cell layer compared to that in Aβ_25–35_-only treated OHSCs. In detail, 0.5 µM of Dex showed a mild protective effect, and over 1 μM, such as 1 μM, 2.5 µM, and 5 μM, all showed similar levels of strong protective effect, suggesting saturation in the effect. For further experiments, such as Western blot, 1 μM, and 2.5 µM were used (Figure 7A).

A significant increase in the abundance of p-tau was observed in the Aβ_25–35_-only treated media. The p-tau production was significantly attenuated in 1 μM Dex-treated Aβ_25–35_-supplemented media, but there was only a decreasing tendency of p-tau production in 2.5 μM Dex-treated Aβ_25–35_-supplemented media. Additionally, p-tau production increased significantly in the 2.5 μM Dex-only-treated media compared to the control (Figure 7B). Dose-dependent neuroprotective effect of Dex was not observed in OHSCs with Aβ_25–35_ and Dex.

PSD-95 levels were considerably low in OHSCs treated with only Aβ_25–35._ PSD-95 levels were significantly restored in Dex-treated Aβ_25–35_ media (1 and 2.5 μM). PSD-95 levels significantly decreased in OHSCs treated with only 2.5 μM Dex (Figure 7B). The intensity of p-CaMKII signals significantly increased in Aβ_25–35_-only treated media and decreased in 1 μM Dex-treated Aβ_25–35_ media. In 2.5 μM Dex-only treated media, p-CaMKII levels were significantly higher than in the control group (Figure 7B).

LC3-II and p62 expression levels were higher in OHSCs treated with only Aβ_25–35_ than in the control. The intensity of LC3-II and p62 staining significantly decreased after treatment with 1 μM Dex. Moreover, LC3-II expression decreased in 2.5 μM Dex-treated Aβ_25–35_ media, confirming improved autophagosome maturation after Dex treatment. The difference between the control and Dex-only treated media was not statistically significant (Figure 7C).

Upregulated expression levels of LAMP2 and cathepsin D were observed in OHSCs treated with only Aβ_25–35_ compared to those in the control (Figure 7C). The intensity of LAMP2 and cathepsin D staining significantly reversed after treatment of 1 μM Dex to Aβ_25–35_ media. LAMP2 and cathepsin D expression did not differ between the control and Dex (1 μM or 2.5 μM)-only treated OHSCs. Taken together, these results indicated that Dex protected neuronal cell death, mitigated dysregulated memory-related proteins by Aβ_25–35_, and regulated autophagy in OHSCs.

To confirm the effect of Dex on autophagy flux, the protein levels of LC3-II or p62 were monitored after treatment with BafA1, an inhibitor of autophagy flux. BafA1 significantly upregulated the levels of LC3-II and p62 (Figure 7D), indicating blockage of autophagosomal maturation. Co-treatment with BafA1 and Dex significantly reduced the intensities of LC3-II and p62 to the control levels in OHSCs (Figure 7D), suggesting Dex as an enhancer of autophagy flux.

## 4 Discussion

The present study demonstrated that Dex mitigated memory impairment and upregulated autophagic flux against Aβ_25–35_-induced toxicity. The recovered behaviors in the Y-maze test and attenuation of dysregulated memory-related proteins p-CaMKII and PSD-95 after Dex treatment showed Dex improved memory impairment induced by Aβ toxicity. This study demonstrated for the first time that Dex reversed increased LC3-II, p62, LAMP2, and cathepsin D levels under Aβ_25–35_ conditions both *in vivo* and in OHSCs, indicating that Dex improved otherwise aberrant autophagic flux against Aβ toxicity. This finding suggested the underlying mechanism and gave insights into the clinical effect of Dex on patients with AD.

Since autophagy plays a critical role in maintaining Aβ homeostasis, Aβ accumulation underlies the impaired autophagic flux in AD pathogenesis (Bordi et al., 2016). Aβ_25–35_, an easily synthesized active form of Aβ (Harkany et al., 2000), has the same effect as the full-length Aβ protein; it has been used to determine autophagy-related mechanisms involved in the development of AD (Fan et al., 2015). As a result, we established the Aβ_25–35_ mice model to confirm the effects of Dex mediated by autophagy. Based on the results of increased p-tau intensity in the Aβ group, we confirmed that the AD model was appropriate. From the results of the decreased intensity of accumulated p-tau in Dex-treated Aβ_25–35_ mice, we hypothesized that Dex has a neuroprotective effect against Aβ_25–35_ toxicity. Several studies have suggested the relationship between Dex and autophagy (Luo et al., 2017; Zheng et al., 2018; Zhang et al., 2021; Kho et al., 2022); however, there is a lack of evidence of the neuroprotective effect of Dex in the AD model. As a result, finding out the neuroprotective mechanism of Dex on AD via autophagy is important to provide evidence of its use in elderly patients with or without degenerative brain diseases.

Memory impairment is a common clinical manifestation of many neurodegenerative diseases, including AD. Intracellular aggregation of Aβ with amyloidosis likely causes neurodegeneration and memory impairment (Nilsson et al., 2013). In this study, the mice in the Aβ group showed decreased behavioral ability in the Y-maze test and deregulated levels of memory-related proteins PSD-95 and p-CaMKII. Decreased PSD-95 intensity in Aβ_25–35_ mice was reversed after Dex administration, indicating that Dex restored synaptic function. PSD-95 is a scaffold protein that enhances synaptic transmission in the cerebral cortex (Béïque and Andrade, 2003); low levels of PSD-95 are associated with the vulnerability of synapses to Aβ (Dore et al., 2021). In addition, Dex treatment reversed the increased p-CaMKII levels in Aβ_25–35_ mice to the control level; p-CaMKII plays an essential role in synaptic plasticity and spatial memory formation in AD (Reese et al., 2011).

Several studies revealed that dysregulated CaMKII exacerbates Aβ formation (Reese et al., 2011; Ghosh and Giese, 2015). Ghosh and Giese (2015) demonstrated that T286 autophosphorylation of CaMKII contributes to NFT formation and cognitive impairment via damaged hippocampal synapses in AD, resulting in neuronal cell death. Reese et al. (2011) showed that redistribution of p-CaMKII leads to decreases in dendrites and synapses instead of increases in the soma of hippocampal CA3 neurons and granule cells of the dentate gyrus. This redistribution of p-CaMKII leads to activity changes from synapse to soma, contributing to synaptic deficits. Moreover, supporting evidence showed that dysregulated PSD-95 and CaMKII activity leads to the loss of synaptic proteins in AD, in line with our results (Ghosh and Giese, 2015). Reduced expression of PSD-95 in neurons is related to neuronal vulnerability mediated by direct activation of the αCaMKII transduction pathway (Gardoni et al., 2002), and upregulated αCaMKII in cornu ammonis areas 1 (CA1) may be a causal factor for cell atrophy in AD (West et al., 1994). However, the result of CaMKII levels in the AD model is controversial. Amada et al. (2005) discovered that reduced αCaMKII in hippocampal CA1 was associated with neuronal loss and cognitive dysfunction in AD. This discrepancy is attributed to the fact that the subcellular localization of dysregulated p-CaMKII is important for synaptic degeneration in the AD hippocampus instead of the total level of p-CaMKII. Notably, our results showed that Dex reversed the upregulation of p-CaMKII in Aβ mice to the control level in the Aβ/D40 group and Aβ/D80 group at all time points (days 1, 3, and 7), especially on days 1 and 7 in the Aβ/D40 group and days 1, 3, and 7 in the Aβ/D80 group, providing evidence that Dex could protect synapses from Aβ toxicity.

Aberrant autophagy leads to an aggravation of Aβ clearance (Geng et al., 2018) and attenuates Aβ secretion in AD (Nilsson et al., 2013). Excessive intracellular Aβ accumulation impairs autolysosome formation and leads to excessive autophagosome accumulation (Ling et al., 2009). Our findings were consistent with previous research that found Aβ induced the accumulation of p-tau, LC3-Ⅰ, and LC3-Ⅱ proteins, indicating a disruption of lysosomal clearance in the autophagic flux pathway (Nixon, 2013). The upregulation of autophagy-related proteins LC3-II and p62 by Aβ_25–35_ could be due to improved autophagic induction or impaired formation of single-membraned autolysosomes, indicating impaired autophagic flux both *in vivo* and in OHSCs. Furthermore, the levels of lysosomal proteins LAMP2 and cathepsin D, which are useful autolysosomal markers for predicting responses to interventions (Mputhia et al., 2019), elevated along with levels of LC3-II and p62 in the Aβ group. This finding also indicated that increased cargo levels of intrinsic lysosomal components were attributed to either impaired induction of autophagy or a defective autophagosome/lysosome fusion pathway.

**FIGURE 8:**
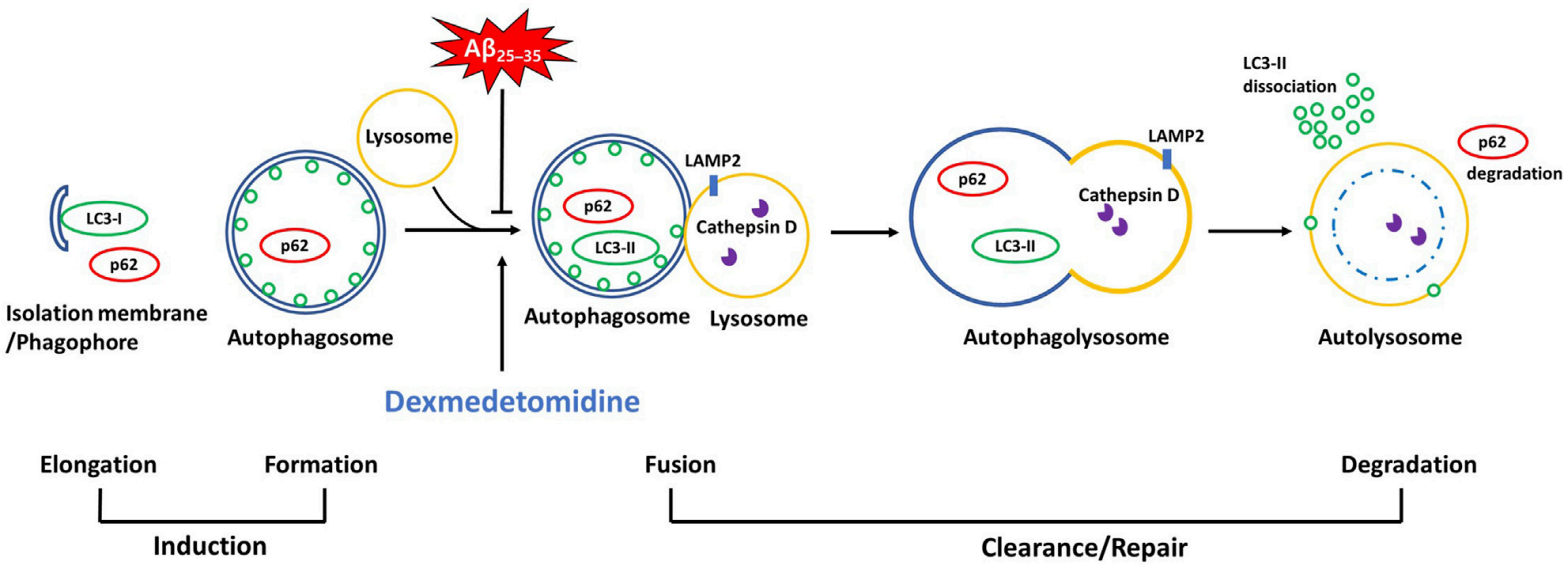
A schematic diagram of the autophagic pathway where Dex improved Aβ_25–35_-impaired autophagic flux There are accumulated autophagic proteins (LC3-II, p62, LAMP2, and cathepsin D) in Aβ_25–35_ groups *in vivo* and *in vitro*, derived by the abbreviated fusion pathway of autophagosomes with lysosomes. Dex attenuated the impaired autophagic flux, as evidenced by decreased levels of the accumulated proteins (LC3-II, p62, LAMP2, and cathepsin D) and co-localized immunoblots.

Dex administration ameliorated Aβ_25–35_-induced upregulation of the autophagy-related proteins LC3-II and p62 and lysosomal proteins LAMP2 and cathepsin D in Aβ_25–35_ mice. Attenuation of increases in LC3-II and p62 after Dex treatment is related to the reduced initial phase of autophagy or improved flow of autophagy, which is called autophagic flux. Previously, Boland et al. (2018) demonstrated that reduced p62 is often related to accelerated autophagic flux as it is a marker for autophagic cargo degradation in autolysosomes (Rahman et al., 2020). Co-localized immunoreactivity of LC3-p62 in the Aβ group was dissociated following Dex treatment, providing evidence of the enhancement of autophagic flux, in which double-membrane autophagosomes were transformed to single-membraned autolysosomes, in consistent with our results of EM. Increased co-localized puncta numbers of LC3-LAMP2 and LC3-cathepsin D following Dex treatment concomitant with a decreased level of the proteins in Western blot showed that impaired autolysosome formation in Aβ mice was alleviated by Dex treatment, in agreement with the previous results that overexpression of cathepsin D improves the degradation of autolysosomes and almost totally restores autophagic flux (Tatti et al., 2012). When Dex treatment was added to BafA1-treated OHSCs to confirm the effect of Dex as an autophagy flux enhancer, BafA1-induced upregulation of LC3-II and p62 was reversed to the control level by Dex, consistent with the results of Aβ treatment. The findings of the present study can be summarized as follows. First, this study confirmed that Dex not only accelerated the initiation of autophagy but also contributed to improving autophagic flux in the Aβ_25–35_ mouse model, confirmed by changes in autophagy-related proteins. (Figure 8). Second, restored autophagic flux might contribute to mitigated memory impairment in Aβ mice after Dex treatment. We confirmed the memory-improving effect of Dex using behavior tests and changes in the expression levels of memory-related proteins.

Moreover, previous studies supported the neuroprotective effects of Dex via autophagy, as presented in this study. Autophagy restoration by Dex contributes to preventing neuronal or organ damage. Zhang et al. (2021) demonstrated that Dex improved autophagic flux integrity; this process regulated and reduced neuronal damage caused by the inflammation response, improving cognitive impairment in mice. Supporting evidence emphasized the organoprotective effects of Dex via autophagy. Dex restored LPS-induced inflammation via improved autophagic flux in the spleen and attenuated certain LPS-induced microRNA modifications in the hippocampus (Kho et al., 2022). Additionally, Zhao et al. (2020) reported that Dex protected the kidney from LPS-induced acute kidney injury by upregulating autophagy. Consistent with the results of previous studies, we confirmed that the neuroprotective effect of Dex in the AD model occurred via improved memory impairment and an upregulated autophagy pathway, which is called autophagic flux.

Based on this evidence, our study findings suggest that Dex could be used as an effective anesthetic adjuvant in the neuroprotective approach because perioperative neurocognitive disorder is one of the severe complications following anesthesia in people with neurodegenerative diseases. Since Aβ deposition is one of the most important causative factors of AD (Karran et al., 2011), mitigating its toxicity by Dex indicates clinical importance. This result was supported by the study that reported a decreased incidence of postoperative cognitive dysfunction in Dex-treated patients with AD and aged people (Zhou et al., 2016). We suggested that Dex might be potentially protective for patients with AD through its effects on the autophagy-related signaling pathway. Accumulated evidence has emphasized the importance of modulating the autophagic pathway to antagonize Aβ toxicity for AD target therapy. Shin et al. (2014) suggested that administering mesenchymal cells enhanced autophagic clearance in AD models both *in vivo* and *in vitro*. Enhancing autolysosome formation by mesenchymal cells might lead to increased neuronal survival against Aβ toxicity. Zhong et al. (2019) also demonstrated neuroprotective effects of Orientin via the enhancement of autophagic flux by modulating LC3-II, p62, and cathepsin D levels in transgenic AD. In line with the previous findings, the present study suggests that regulating the autophagy pathway may play a role in offsetting Aβ toxicity in AD. Based on the neuroprotective effect of Dex, we proposed the mechanism of Dex-mediated protection against Aβ_25–35_-induced neurotoxicity by investigating its role in the autophagy-lysosomal pathway, providing a basis for using Dex as an anesthetic adjuvant in patients with neurodegenerative diseases, particularly AD.

Our study did not show better neuroprotective effects in the Aβ/D80 group of *in vivo* than the Aβ/D40 group, and the Aβ + Dex 2.5 group of OHSCs than the Aβ + Dex 1 group. Moreover, the D80 group seemed to have some negative effects, in some parts. For example, D80 showed increased levels of p-tau and decreased tendencies of PSD-95, although most of them were not significant, with significance observed only on day 3 for p-tau. It might be attributed to hemodynamic side effects (e.g., bradycardia and hypotension) of Dex, similar to the studies showing high doses of Dex did not enhance its neuroprotective properties (Liu W. J. et al., 2016; Pan et al., 2016). However, the Y-maze test showed no negative effects in the D80 group, and Aβ/D80 cohort also showed reversed tendencies against Aβ-induced toxicity in most experiments. So, it could not be concluded that 80 μg/kg of Dex is neuroprotective or neurotoxic at this time. In the future, further research will be required to examine the effects of Dex at various doses.

In conclusion, the present study demonstrated that Dex could attenuate memory impairment in Aβ_25–35_ mice. We confirmed the neuroprotective mechanism of Dex, which is linked to its alleviation of impaired autophagic flux. These findings suggest that Dex could be clinically used as a neuroprotective adjuvant in anesthesia for patients with AD with caution in dose.

## Data Availability

The original contributions presented in the study are included in the article/Supplementary Material, further inquiries can be directed to the corresponding authors.

## Abbreviations

AD, Alzheimer’s disease; Aβ25–35, amyloid β-protein fragment 25–35; Dex, dexmedetomidine; i.c.v., intracerebroventricular; IP, intraperitoneal; LPS, lipopolysaccharide; NFT, neurofibrillary tangle; LC3, microtubule-associated protein light chain 3; LAMP2, lysosome-associated membrane protein 2; Nrf2, nuclear factor erythroid 2-related factor; p-CaMKII, phosphorylated Ca2+/calmodulin-dependent protein kinase II; PSD-95, postsynaptic density-95; OHSC, organotypic hippocampal slice culture; LORR, loss of righting reflex; PBS, phosphate-buffered saline; PI, propidium iodide; SEM, standard error of the mean; CA3, cornu ammonis areas 3; BafA1, bafilomycin A1.
