# Supplementary material for: Neuroprotective effect of dexmedetomidine on autophagy in mice administered intracerebroventricular injections of Aβ25–35
Source: Front Pharmacol. 2023 Aug 17;14:1184776. doi: 10.3389/fphar.2023.1184776 (PMC10469611; doi:10.3389/fphar.2023.1184776)
Supplement: Supplementary file 1 [file DataSheet2.PDF]

Supplemental Table 2. Secondary antibodies for immunofluorescence staining and Western blotting

Secondary antibodies	Label	Source organism	Company	Application
anti-rabbit IgG	Alexa fluor 555	Goat	Molecular probes, Eugene, OR	IF (1:250)
anti-mouse IgG	Alexa fluor 555	Goat	Molecular probes, Carlsbad, CA	IF (1:250)
anti-mouse IgG	FITC	Goat	CHEMICON international, Temecula, CA	IF (1:250)
anti-rabbit IgG	FITC	Goat	CHEMICON international, Temecula, CA	IF (1:250)
anti-rabbit IgG	HRP	Goat	Jackson ImmunoResearch, Grove, PA	WB (1:1000)
anti-mouse IgG	HRP	Goat	Jackson ImmunoResearch, Grove, PA	WB (1:1000)

FITC; fluorescein isothiocyanate, HRP; Horseradish peroxidase, IF; immunofluorescence staining, WB; Western blot analysis.
