# Supplementary material for: Neuroprotective effect of dexmedetomidine on autophagy in mice administered intracerebroventricular injections of Aβ25–35
Source: Front Pharmacol. 2023 Aug 17;14:1184776. doi: 10.3389/fphar.2023.1184776 (PMC10469611; doi:10.3389/fphar.2023.1184776)
Supplement: Supplementary file 2 [file DataSheet1.PDF]

Supplemental Table.1 Primary antibodies for immunofluorescence staining and Western blotting

Primary antibody	Specificity	Source organism	Company	Application
p-tau (Ser 396)	Monoclonal	Mouse	Santa Cruz Biotechnology, Santa Cruz, CA	IF (1:200)<br>WB (1:200)
NeuN	Polyclonal	Rabbit	Abcam, Waltham, MA	IF (1:200)
PSD-95 (22B1)	Monoclonal	Mouse	Santa Cruz Biotechnology, Santa Cruz, CA	IF (1:500)<br>WB (1:200)
p-CaMKII (Thr 286)	Monoclonal	Mouse	Santa Cruz Biotechnology, Santa Cruz, CA	WB (1:500)
LC3A	Polyclonal	Rabbit	SIGMA-ALDRICh, St. Louis, MO	IF (1:200)
LC3B	Polyclonal	Rabbit	ABGENT. San Diego, CA	IF (1:200)<br>WB (1:2000)
p62	Polyclonal	Rabbit	SIGMA-ALDRICh, St. Louis, MO	IF (1:500)<br>WB (1:2000)
LAMP2	Polyclonal	Rabbit	Abcam, Waltham, MA	WB (1:2000)
Actin	Monoclonal	Mouse	Santa Cruz Biotechnology, Santa Cruz, CA	WB (1:1000)

p-tau; phosphorylated-tau, PSD-95; postsynaptic density-95, p-CaMKII; phosphorylated alpha isoform of calcium/calmodulin-dependent protein kinase II, LC3A/B; microtubule-associated protein 1 light chain 3 beta, LAMP2; lysosomal associated membrane protein-2, IF; immunofluorescence staining, WB; Western blot analysis.
